# BFF-piRNA-seq reveals conserved processing hotspots in flies and mice

**DOI:** 10.21203/rs.3.rs-11307628/v1

**Published:** 2026-10-26

**Authors:** Astrid Haase, Markus Hafner, Parthena Konstantinidou, Franziska Ahrend, Brian Galletta, Angel Jaimes, Dimitrios Anastasakis, Nasser Rusan

**Affiliations:** NIH/NIDDK; NIAMS/NIH; NIH/NHLBI; NIDDK/NIH; University of Crete; NIH/NHLBI

## Abstract

PIWI-interacting RNAs (piRNAs) protect germline genome integrity and fertility, but quantitative profiling of mature PIWI-bound RNAs remains technically challenging. Here we develop BFF-piRNA-seq, which ligates sequencing adapters directly to RNAs on immobilized PIWI complexes, eliminating preparative polyacrylamide gel electrophoresis and associated size-selection bias. Split-sample comparisons of on- and off-PIWI ligation show highly concordant length distributions and no detectable terminal sequence bias, and the method reproducibly profiles distinct PIWI-piRNA complexes in mouse and *Drosophila*. Eliminating gel selection recovers a broader population of *Drosophila* Piwi-piRNAs than conventional workflows. Across flies and mice, quantitative profiles reveal a conserved landscape across piRNA clusters in which narrow hotspots generate abundant families of sequence-related piRNAs with 5′ microheterogeneity. Together, these data establish BFF-piRNA-seq as an accessible, quantitative approach for profiling bona fide piRNAs and uncover conserved principles governing piRNA selection during biogenesis and maturation.

## Introduction

PIWI-interacting RNAs (piRNAs) are central to a conserved RNA-guided immune system that protects germline genome integrity from the deleterious activity of endogenous retroviruses and other mobile genetic elements (transposons)^1-4^. These ~ 20–40 nucleotide long non-coding RNAs guide PIWI proteins to silence transposons by establishing epigenetic restriction and degrading their transcripts in pre-meiotic germ cells. In mammals, an additional meiotic piRNA pathway has been implicated in regulating gene expression during spermatogenesis. Disruption of either pathway -through mutations in PIWI proteins or essential biogenesis or effector factors- causes defective germ cell development and sterility across animals, and has been linked to human infertility^5^.

piRNAs represent the most diverse class of eukaryotic small silencing RNAs, comprising millions of sequences derived from long precursor transcripts^3^. While the core machinery of piRNA biogenesis and function is conserved from flies to humans, the sequences of piRNA precursors and mature piRNAs evolve rapidly in response to the shifting transposon landscape^4^. Their lack of sequence conservation and the extreme differences in individual piRNA abundance, create major challenges for piRNA analyses and functional interpretation^2^. Although substantial progress has established a conceptual framework for piRNA biology, key mechanistic features of piRNA processing, the principles of piRNA-target recognition, and the functions of individual piRNAs remain largely elusive^6^.

Accurate identification and quantitative profiling of bona fide piRNAs are central to piRNA research^7,8^. Conventional workflows for sequencing PIWI-bound small RNAs typically rely on preparative polyacrylamide gel electrophoresis (PAGE) to separate ligated products from excess adapters; gel excision can be technically demanding and can introduce user-dependent size-selection bias^9,10^. To simplify rigorous piRNA profiling while reducing size-selection bias, we developed BFF-piRNA-seq (Bona fide, gel-Free, label-Free piRNA sequencing). Our streamlined method leverages the defining feature of piRNAs as PIWI-interacting RNAs by directly ligating a 3′ adapter to purified, immobilized PIWI-piRNA complexes (‘on-PIWI-ligation’). This approach eliminates preparative PAGE purification and labeling while preserving key features required for quantitative piRNA profiling, including adapters with randomized terminal nucleotides and unique molecular identifiers (UMIs) that minimize ligation bias and enable accurate molecular quantification, resulting in highly reproducible, nucleotide-resolution piRNA data. By reducing handling and size-selection constraints while improving quantitative fidelity, BFF-piRNA-seq broadens access to researchers in development, fertility, and evolution. Using BFF-piRNA-seq, we define a conserved logic of piRNA biogenesis in flies and mice, whereby narrow processing hotspots generate families of sequence-related piRNAs with 5′ microheterogeneity.

BFF-piRNA-seq is built around a single organizing principle: capture and tag bona fide piRNAs while they remain bound to their PIWI partner. Specifically, 3′ adapters are ligated directly to piRNAs on immobilized PIWI–piRNA complexes, allowing excess adapter to be removed by stringent washing rather than preparative PAGE. Quality standards of established PAGE- and label-dependent small-RNA sequencing methods are retained: randomized terminal nucleotides minimize ligation bias, while 10-nt unique molecular identifiers (UMIs) enable molecular counting and removal of PCR duplicates^11-13^. We benchmarked BFF-piRNA-seq across MIWI, MILI, and *Drosophila* Piwi, including direct on- versus off-PIWI ligation controls and biological replicates. The method reproduces established piRNA features while recovering a broader *Drosophila* Piwi-bound repertoire and reveals a conserved hotspot-dominated organization of mature PIWI-bound piRNAs in flies and mice.

## Results

### On-PIWI ligation enables gel-free piRNA sequencing

To sequence bona fide piRNAs without preparative PAGE size selection, we exploit their defining feature, their interaction with PIWI proteins^7^. Unlike long RNA-sequencing methods that quickly arrive at cDNA and amplification, small RNA-seq methods must first ligate adapters to both RNA ends (Fig. 1a)^9^. Because adapters and small RNAs are similar in size, adapter–adapter dimers and concatemers are hard to avoid. Prior solutions devised pre-adenylated 3′ adapters to avoid concatemerization but typically required urea-PAGE purification to remove excess adapters before subsequent steps^9^. Here, we perform 3′ adapter ligations directly on immobilized PIWI-piRNA complexes, then remove unligated adapters by stringent washes rather than urea-PAGE purification (Supplementary Fig. 1a). This “on-PIWI” workflow eliminates the need for labeling and PAGE purification steps used in legacy protocols^9,10^.

We benchmarked BFF-piRNA-seq on the well-characterized MIWI (PIWIL1)- and MILI (PIWIL2)-bound piRNA populations from adult mouse testes and found that it quantitatively captures unbiased sequence and length profiles. To directly assess potential bias introduced by on-PIWI 3′ adapter ligation, we compared data generated by ligating adapters either directly on immobilized PIWI-piRNA complexes (“on-PIWI”) or after elution of the same captured piRNAs (“off-PIWI”) (Fig. 1b). Both workflows produced indistinguishable piRNA length distributions, accurately recovering the expected length distributions peaking at 30-nt for MIWI piRNAs and 27-nt for MILI piRNAs (Fig. 1c)^7,14,15^. We then asked whether on-PIWI ligation introduces sequence bias at ligation junctions. Because both adapters contain randomized terminal nucleotides (N) at their ligating ends (Fig. 1d), nucleotide-specific biases were minimal and similarly low in both on-PIWI and off-PIWI libraries. Although off-PIWI ligation without PAGE purification was sufficient for generating control libraries, we do not recommend it as a standard workflow because it produces substantial adapter-adapter dimers and background degradation fragments from abundant cellular RNAs, including rRNAs, tRNAs, and snoRNAs **(Supplementary Fig. 1b).** Together, these results show that on-PIWI adapter ligation preserves native piRNA composition and enables quantitative sequencing of bona fide piRNAs without detectable sequence or length bias.

Next, we benchmarked BFF-generated pachytene MIWI- and MILI-bound piRNAs against standard datasets with respect to length distribution, genomic annotation, established processing signatures, and origin from piRNA-producing genomic loci (piRNA clusters). Consistent with published datasets, BFF-piRNA-seq recovered MIWI-bound piRNAs with the expected peak at 30 nt and an overall length distribution spanning ~ 25–35 nt (Fig. 2a)^16^. Most piRNAs mapped uniquely to the genome and predominantly originated from intergenic regions, with transposon-derived sequences underrepresented relative to their genomic abundance, as expected for pachytene piRNAs (Fig. 2b**).** These piRNAs also showed the characteristic 1U bias, a hallmark of primary piRNA biogenesis (Fig. 2c) ^2,4,17^. As reported previously, the vast majority of MIWI-bound piRNAs mapped to discrete pachytene piRNA clusters, with the top ~ 90 clusters accounting for more than 90% of all MIWI piRNAs (Fig. 2d **and Supplementary Fig. 2a,b)**^7,14,18,19^. Across three individual mice, BFF-generated MIWI datasets showed highly concordant length distributions, sequence features, and genomic annotations, underscoring both technical reproducibility and biological robustness. Parallel analyses of MILI-bound piRNAs yielded the same result, confirming that BFF-piRNA-seq robustly recovers the established biological features of both MIWI and MILI piRNA populations (Fig. 2e-h and Supplementary Fig. 2c,d). Together, these analyses demonstrate that on-PIWI ligation shows no detectable length or sequence bias, and that BFF-piRNA-seq faithfully preserves the defining features of bona fide piRNAs.

### Hotspots generate families of related piRNAs

Closer inspection of the most productive piRNA processing sites revealed narrow peaks composed of single or clustered, highly abundant piRNA 5′ start sites (Fig. 5d and Supplementary Fig. 5d). The sub-piRNA-length spacing between these 5′ start sites is consistent with alternative cleavage positions being used on different precursor molecules. Closely spaced starts therefore define narrow hotspot intervals that are repeatedly sampled across the mature piRNA population. BFF-piRNA-seq reveals that processing is concentrated within these narrow precursor segments, whereas the exact 5′ position shows microheterogeneity, generating families of piRNAs with closely related sequences but distinct 5′ ends.

To begin defining features of processing hotspots that may underlie their activity, we analyzed the 50 most abundant piRNA 5′ ends at the beginning of flamenco. Although these sites accounted for only ~ 5% of unique piRNA sequences within this interval, they generated more than 20% of all piRNA molecules. Sequence-logo analysis, including flanking nucleotides, recovered the well-established 1U bias but revealed no additional sequence preferences or motifs upstream, within, or downstream of hotspot piRNAs (Fig. 5e and Supplementary Fig. 5e). Notably, the 1U bias was stronger among the top 50 hotspot piRNAs than among adjacent, less abundant piRNAs, consistent with a broader positive relationship between piRNA abundance and features of primary processing^26^.

### Mature hotspot piRNAs lack flanking phasing patterns

Previous studies reported string-like patterns of piRNAs at select positions, raising the possibility that processing of one piRNA may initiate generation of a downstream piRNA^29,32^. To assess whether processing hotspots are associated with in-phase piRNA string patterns, we analyzed the distribution of piRNA 5′ start sites positioned approximately one piRNA length (~ 27 nt) upstream and downstream of the most abundant piRNAs. To avoid ambiguity due to multimapping sequences, we first focused on the well-mappable 5′ region of flamenco and a prominent site within cluster 42AB (Fig. 6a and Supplementary Fig. 6a,b), and then extended our analyses genome-wide across multiple well-mappable cluster segments (Fig. 6b and Supplementary Fig. 6c,d). Neither analysis revealed a consistent enrichment of piRNA starts at one-piRNA-length intervals. Instead, mature hotspot populations were locally confined and showed narrow 5′-end microheterogeneity.

As an orthogonal test, we examined the global positional relationship between piRNA 5′ starts and 3′ ends, a metric previously suggested to reflect phased piRNA production^32^. As reported previously, piRNA 5′ starts were enriched at positions coinciding with 3′ termini. However, such enrichment could arise either from coupled 3′/5′ processing during phased biogenesis or from the shared +1U/1U sequence preference at piRNA 3′ and 5′ ends. To distinguish between these possibilities, we stratified piRNAs by their 5′ nucleotide and repeated the analysis separately for 1U and non-1U piRNAs (1A, 1C, or 1G). If the overlap between 5′ starts and 3′ ends were driven by shared sequence preference, the signal should be restricted to 1U piRNAs and absent from non-1U piRNAs. By contrast, if it reflected simultaneous generation of both ends during phased processing, it should be present in both groups. We detected a positive correlation only for 1U piRNAs, whereas non-1U piRNAs showed no positional concordance between 5′ and 3′ ends. Thus, in the mature piRNA population, the apparent 3′/5′ alignment is explained by the shared +1U/1U sequence preference and does not provide evidence for phased piRNA biogenesis (Fig. 6c).

Overall, BFF-piRNA-seq reveals key features of primary piRNA biogenesis in flies. By recovering the broader population of Piwi-bound piRNAs, the method shifts length distributions and relative abundances toward patterns more closely resembling those observed in mammals^26,27^. Although the canonical 1U bias and origin from known piRNA clusters are reaffirmed, the unrestricted and quantitative datasets show that mature Piwi-bound piRNAs are concentrated within narrow hotspots that generate families of closely related piRNAs. The clustered microheterogeneity of 5′ starts within these narrow windows is consistent with biogenesis preferentially sampling selected precursor segments rather than imposing a single precise 5′ position.

### Hotspot organization is conserved in mouse

Intrigued by the distinct and robust processing patterns observed in flies, we re-evaluated processing features of mammalian pachytene piRNAs. BFF-MIWI-piRNA-seq from adult mouse testes revealed rugged piRNA profiles across piRNA clusters, exemplified by piRNAs originating from the major piRNA clusters pi18 and pi17 (Fig. 7a and Supplementary Fig. 7a)^33,34^. As in Drosophila, piRNA patterns were marked by narrow peaks and deep valleys with peak-to-average ratios (PAR) around 11, indicating that the most abundant piRNAs are 11-fold more abundant than the average piRNA from the same precursor (Fig. 7b and Supplementary Fig. 7b). The genome-wide peak-to-valley patterns were highly reproducible between mice, underscoring biological robustness and technical reproducibility (Fig. 7c and Supplementary Fig. 7c–e).

Closer inspection of peak regions revealed narrow processing hotspots with one or several dominant 5′ start sites, similar to the patterns observed in flies (Fig. 7d). These hotspots produced highly abundant, overlapping piRNAs, indicating that mature piRNA output is concentrated at preferred precursor intervals while permitting multiple nearby 5′ positions. Many of the most abundant 5′ start sites clustered within ~ 50-nt windows (Fig. 7d, bottom). Because closely spaced start sites cannot all arise from the same precursor molecule, their organization is consistent with alternative cleavage positions being used across precursor molecules, generating sequence-related piRNAs with 5′ microheterogeneity (model, Fig. 7e).

The most abundant 5′ ends showed a stronger 1U bias, consistent with a conserved enrichment of 1U over 1A-, 1C-, and 1G-piRNAs during primary piRNA biogenesis (**Supplementary Fig. 7f,g**)^26^. We did not detect positional sequence preferences or a consensus motif upstream, within, or downstream of hotspot piRNAs, suggesting that local primary sequence alone does not explain hotspot position^35,36^. Taken together, the robust piRNA profiles generated by BFF-piRNA-seq reveal a conserved biogenesis landscape in flies and mice in which processing is concentrated at discrete hotspots that produce highly abundant piRNAs. What directs the piRNA processing machinery to these stable hotspots, and why extensive intervening precursor regions contribute only sparsely to the mature piRNA pool, remain important open questions.

## Discussion

BFF-piRNA-seq couples biochemical identification of bona fide piRNAs to quantitative library preparation by performing the first adapter-ligation step directly on immobilized PIWI–piRNA complexes. Eliminating preparative PAGE reduces handling and removes user-defined size cutoffs while randomized adapter termini and UMIs support bias reduction and molecular counting. The method is reproducible across biological replicates and distinct PIWI proteins and, in *Drosophila*, recovers a broader Piwi-bound RNA population than conventional size-selected workflows. This technical improvement therefore changes the biological measurement rather than merely simplifying sample preparation.

Across flies and mice, BFF-piRNA-seq revealed a conserved organization of mature PIWI-bound piRNA populations dominated by discrete hotspots rather than broadly uniform representation across long precursors. Individual hotspots comprise one or a few preferred 5′ start positions and generate families of abundant, sequence-related piRNAs with variable 5′ ends. This 5′ microheterogeneity is consistent with primary processing repeatedly sampling narrow precursor intervals rather than fixing a single precise 5′ start site. Because BFF-piRNA-seq measures mature PIWI-bound piRNAs, it focuses on functional PIWI-piRNA complexes required for piRNA:target recognition. In contrast to miRNA-like, seed-based recognition, which depends on strict 5′ register, target engagement through extended complementarity is more compatible with limited variability at the 5′ end^37-39^. The sequence variation observed among hotspot-derived piRNAs is therefore consistent with emerging models in which piRNAs act through broader complementarity^38,40-42^.

The overrepresentation of hotspot-derived piRNAs, together with deep troughs across intervening precursor regions, further indicates that mature piRNA output is highly selective. RNA-binding proteins, RNA structure, and slicing have each been implicated in precursor choice and processing specificity^30,35,43,44,45^. BFF-piRNA-seq now provides a practical framework to reproducibly define productive hotspot regions at high resolution and to identify the features that recruit the processing machinery to these sites. More generally, our data suggest that only a subset of precursor sequence contributes strongly to the mature piRNA population. Focusing piRNA processing at a limited number of productive sites may promote effective piRNA output while minimizing accumulation of potentially harmful species, and may help explain how long precursor loci remain evolvable while preserving essential function.

## Methods

### Materials availability

Further information and requests for resources and reagents should be directed to and will be fulfilled by the corresponding author, Astrid D. Haase (astrid.haase@nih.gov).

### Experimental models

#### Drosophila ovarian somatic cells

Ovarian somatic sheath cells (OSC) expressing endogenously tagged Piwi (FLAG-HA-(FH-)Piwi) were previously described by *Meng Q, et al.* ^46^ and are available from the Drosophila Genomics Resource Center (DGRC stock number: 322).

#### Drosophila melanogaster

Drosophila ovaries were obtained from flies generated by crossing a UASp-FLAG-HA-Piwi strain (genotype: w[*] (BDSC #83080); P{w[+ mC]=UASp-FLAG-HA-Piwi[wt]}/CyO) with either a tj-Gal4 strain (genotype: w[*]; w[+] tj-Gal4/ CyO) or a nos-Gal4 strain (genotype: UAS-dcr2; nos-gal4/CyO; Dr/ TM6B, Tb[1]). Stocks are available from the Bloomington Drosophila Stock Center.

### Mice

Mouse testes were dissected from adult male wild-type C57BL/6 mice at 16 weeks of age for the main datasets and at 8 weeks of age for the on- versus off-PIWI ligation controls. Mice were maintained and euthanized in accordance with National Institutes of Health guidelines (NIH/NIDDK animal-care committee approval: protocol # K009-LCMB-26).

### BFF-piRNA-seq library preparation (Step-by-Step Protocol available as Supplemental file)

piRNA datasets in this study were prepared using the BFF protocol. A detailed step-by-step protocol and troubleshooting guide are provided in the Supplementary Methods. The following PIWI-piRNA complexes were immunoprecipitated: FLAG-HA-tagged Piwi (FH-Piwi) from OSC cells and Drosophila ovaries using anti-FLAG M2 magnetic beads (Sigma, M8823), MIWI from adult mouse testes using Protein A Dynabeads (Invitrogen, 10001D) coupled with rabbit α-MIWI antibody (Cell Signalling, 2079) according to the manufacturer’s instructions. MILI from adult mouse testes using Protein A Dynabeads (Invitrogen, 10001D) coupled with rabbit α-MILI antibody (Cell Signalling, 5940) according to the manufacturer’s instructions. The defining feature of BFF is the 3′ adapter ligation to immobilized PIWI-piRNA complexes. Following 3′ ligation, excess unligated adapter is removed by washing, and 3′ ligated piRNAs are extracted using Trizol and purified using the DirectZol RNA miniprep kit (Zymo Research, R2051) before 5′ adapter ligation and cDNA synthesis. To minimize PCR duplicates and ensure quantitative accuracy of the generated data, we employed two complementary strategies. First, before library barcoding a pilot PCR was performed to determine the minimum cycle number required. This step prevents overamplification and waste of sequencing reads on PCR artifacts. Second, all libraries prepared for this study contain 10 unique molecular identifiers (UMIs): 8 at the 5′ end and 2 at the 3′ end of piRNAs. UMIs enable the computational removal of any remaining PCR duplicates after sequencing, ensuring accurate quantification of individual piRNA abundances. Libraries were sequenced by the National Institute of Arthritis and Musculoskeletal and Skin Diseases Genomic Technology Section using a NextSeq 2000 (Illumina) and obtaining 75 nt, single-end reads.

#### Library-preparation enzymes and purification reagents

For lysis and PIWI immunoprecipitation, samples were prepared with cOmplete Mini EDTA-free protease inhibitor cocktail (Sigma-Aldrich, 04693159001). The BFF library workflow used T4 RNA ligase 2 truncated K227Q (New England Biolabs (NEB), M0351) for 3′ adapter ligation and T4 RNA ligase 1 (NEB, M0437M) for 5′ adapter ligation, with SUPERase•In RNase inhibitor (Invitrogen, AM2694). Reverse transcription used SuperScript IV (Invitrogen, 18090010); PCR used Platinum Taq DNA polymerase (Invitrogen, 15966005) and dNTP mix (10 mM each; NEB, N0447).

After on-PIWI ligation, RNA was extracted using TRIzol and Direct-zol RNA Miniprep (Zymo Research, R2051). Subsequent purification steps used Oligo Clean & Concentrator (Zymo Research, D4060) and DNA Clean & Concentrator-5 (Zymo Research, D4004). Adapter-dimer products were separated using 3% agarose Pippin Prep cassettes (Sage Science, CDP3010); libraries were assessed using TapeStation D1000 reagents (Agilent, 5067–5582 and 5067–5583). Details of reaction conditions, purification, PCR cycling, and troubleshooting are provided in the Supplementary Methods (step-by-step BFF-piRNA-seq protocol).

#### BFF adapters and primers

The 3′ adapters were 5′ pre-adenylated and 3′ blocked and carried two randomized terminal nucleotides (N). The 5′ DNA–RNA hybrid adapter provided eight additional randomized nucleotides, giving 10 unique molecular identifier (UMI) positions per library. All oligonucleotide sequences are written 5′ to 3′, with supplier modification notation retained as in the original reagent list:

##### 3′ adapter 29.01

/5rApp/NNTGACTGTGGAATTCTCGGGTGCCAAGG/3AmMO/

##### 3′ adapter 29.02

/5rApp/NNACACTCTGGAATTCTCGGGTGCCAAGG/3AmMO/

##### 3′ adapter 29.03

/5rApp/NNACAGAGTGGAATTCTCGGGTGCCAAGG/3AmMO/

##### 3′ adapter 29.04

/5rApp/NNGCGATATGGAATTCTCGGGTGCCAAGG/3AmMO/

##### 5′ DNA–RNA adapter

/5AmMC6/GTTCAGAGTTCTACAGTCCGACGArUrCrNrNrNrNrNrNrNrN

##### Reverse-transcription (RT) primer

GCCTTGGCACCCGAGAATTCCA

##### 5′ short PCR primer

CTTCAGAGTTCTACAGTCCGACGA

##### 5′ long PCR primer

AATGATACGGCGACCACCGAGATCTACACGTTCAGAGTTCTACAGTCCGA

##### 3′ index PCR primer (P1-A1 example)

CAAGCAGAAGACGGCATACGAGATGTCGGTAAGTGACTGGAGTTCCTTGGCACCCGAGAATTCCA

The 3′ adapters (29.01–29.04) contain distinct internal sample barcodes. The sequence shown for the 3′ index PCR primer corresponds to P1-A1 from NEBNext Multiplex Oligos 96. Additional 3′ index primer sequences and barcode combinations are provided in the Supplementary Methods protocol.

### Published piRNA datasets

The following public piRNA datasets generated by standard protocols that employ urea-PAGE purification were downloaded and used for comparison: MIWI-piRNAs from sorted mouse spermatocytes (SRR7760307) ^47^ and Piwi-piRNAs from *Drosophila* OSC cells (SRR12430866, SRR12430869, SRR12430863) ^26^.

The reference mouse MIWI-IP and MILI-IP datasets are SRR7760307 and SRR7760331 (BioProject PRJNA421205; Gainetdinov et al., 2018), respectively. Published Drosophila ovarian somatic-cell Piwi-piRNA datasets are SRR12430866, SRR12430869, and SRR12430863 (BioProject PRJNA656552; Genzor et al., 2021). Sequencing data generated in this study for Drosophila germline, follicle cells and OSCs, and adult mouse MIWI and MILI complexes, were deposited under GEO accession GSE313113.

### On- versus off-PIWI ligation controls

To establish the efficiency of on-PIWI 3′ adapter ligation, we performed a direct comparison with conventional in-solution ligation. MIWI and MILI immunoprecipitation samples from 8-week-old mice (n = 3) were split into two portions: one underwent 3′ adapter ligation on immobilized MIWI- or MILI–piRNA complexes (on-PIWI ligation), while piRNAs in the other portion were eluted from complexes and ligated in solution (off-PIWI ligation). After RNA purification, the two fractions were pooled back together for 5′ adapter ligation, cDNA library preparation and were distinguished computationally using distinct 3′ adapter indices. We compared the length distributions and terminal nucleotide identities of the two methods (see Fig. 1).

### Piwi protein quantification

We estimated cellular Piwi protein abundance using our previously characterized endogenously tagged FLAG-HA-Piwi OSC line (OSC:eFH-piwi) ^46^. We previously established that OSC:eFH-Piwi is heterozygous and that eFH-Piwi expression matches endogenous Piwi allele expression levels. Fluorescent western blotting was performed as previously described in ^46^ with the following modifications. After transfer, membrane was incubated with 1:1000 mouse anti-FLAG M2 antibody (Sigma, F3165) overnight at 4°C. The following day, membrane was washed three times with TBST before incubation with 1:10000 IRDye 800 goat anti-mouse secondary antibody (926-32210, LI-COR) for 50 min at room temperature in darkness. Membrane was washed five times with TBST, and fluorescence was detected using Odyssey CLx imaging system (LI-COR). To quantify eFH-Piwi molecule numbers, we used serial dilutions of amino-terminal FLAG-BAP fusion protein (MilliporeSigma, P7582) as molecular standards. To enhance quantification reliability, we analyzed multiple cell concentrations (ranging from 12,500 to 100,000 cells) from two independent pellets (see Supplementary Data 1. PIWI quantification raw data and calculations).

### Isolation of somatic and germline Piwi-piRNAs

To isolate follicle- and germline-specific Piwi-piRNA complexes from Drosophila ovaries, we expressed a FLAG-HA-tagged Piwi (FH-Piwi) protein driven by either the somatic marker tj or the germline marker nos. This was achieved by crossing a UASp-FLAG-HA-Piwi strain (genotype: w[*]; P{w[+ mC]=UASp-FLAG-HA-Piwi[wt]}/CyO) with either a tj-Gal4 strain (genotype: w[*]; w[+] tj-Gal4/ CyO) or a nos-Gal4 strain (genotype: UAS-dcr2; nos-gal4/CyO; Dr/ TM6B, Tb[1]). Following eclosion, adult females of the desired genotypes were moved to standard media supplemented with yeast paste for 2–3 days prior to dissection. A subset of dissected ovaries was used to verify tissue-specific expression of FH-Piwi by immunofluorescence, and the rest served as input for BFF-piRNA-seq (20–30 ovaries).

### Immunofluorescence microscopy

Ovaries were dissected in Drosophila S2 media (Gibco, Thermo Fisher Scientific, 21720001). Dissected ovaries were fixed for 30 min in 9% formaldehyde in phosphate-buffered saline (PBS) with agitation. At the beginning of the fixation period ovaries were disrupted with a microfuge pestle and short vortex pulses to free ovarioles from the ovary. Following fixation, samples were rinsed three times in PBS + 0.3% Triton X-100 (PBST) and then stored at 4°C (less than 1 week). Samples were blocked for approximately 6 hours in 5% normal goat serum (Millipore Sigma, G9023) in PBST for approximately 6 hours. Blocked samples were incubated overnight with primary antibodies (1:500 rabbit anti-PIWI (Haase lab ^28^); 1:250 mouse α-HA (Biolegend, 901501)) in block at 4°C with agitation then washed the following morning in PBST (three times for 10 min with agitation). Secondary antibodies (1:1000; anti-mouse Alexa Fluor 488, Cat # A11029 and anti-rabbit Alexa Fluor 647, Cat# A21244, Thermo Fisher Scientific) in block were incubated with samples for 3 h at room temperature with agitation. Samples were washed three times for 10 min in PBST, then mounted in a drop of Aquapolymount (Polysciences, 18606-20) under a No. 1.5 coverslip. Images were acquired on a Nikon Eclipse Ti2 microscope (Nikon Instruments) with a 100×/1.49 total internal reflection fluorescence oil immersion objective; W1 spinning disk confocal head (Yokogawa, Life Science); 488- and 647-nm lasers; and a Prime BSI cMOS camera (Teledyne Photometrics). Image analysis and processing was performed with Fiji (ImageJ; National Institutes of Health).

### Computational analysis

#### Software and analysis tools

Sequence preprocessing used cutadapt v4.4 (https://github.com/marcelm/cutadapt), and genome alignment used STAR v2.7.10b (https://github.com/alexdobin/STAR). Coverage and replicate comparisons used deepTools (https://github.com/deeptools/deepTools). Statistical and genomic analyses were carried out in R (version 4.2.1 or later; https://www.r-project.org/) using piRNA Cluster Builder (PICB) v1.2.0 (https://bioconductor.org/packages/PICB/), DESeq2 (https://bioconductor.org/packages/DESeq2/), and packages specified below. Microscopy images were examined with ImageJ/Fiji (https://github.com/imagej/ImageJ). Custom meta-analysis code is supplied as Supplementary Software 1.

### Processing of piRNA-sequencing data

Raw sequencing reads were processed as follows. First, the 3′ adapter constant sequence (i.e., without the UMIs) was trimmed using cutadapt ^48^. Note that ligation control experiments with different 3′ adapter indices were also demultiplexed during trimming by cutadapt (cutadapt -a On_PIWI=TGACTGTGGAATTCTCGGGTGCCAAGG -a In_solution=ACACTCTGGAATTCTCGGGTGCCAAGG - -overlap 10 -m 29 -o trimmed_{name}.fastq.gz Initial.fastq.gz). For libraries containing UMIs (all BFF libraries and the publicly available *Drosophila* datasets), PCR duplicates were eliminated by collapsing trimmed reads with identical sequences followed by trimming of UMIs using cutadapt (cutadapt -u 8 -u -2). Abundant cellular RNAs (rRNAs, tRNAs, miRNAs, snRNAs, snoRNAs) were removed by annotation and the remaining reads were mapped to the reference genome (Dm6 for *Drosophila* and mm10 for mouse libraries) using STAR ^49^ and allowing up to 1 mismatch and 100 alignments per read. Spliced alignments were suppressed by using indexes generated without annotation (GTF) files and applying the STAR parameter -- alignIntronMax 1.

### Analysis of mapped piRNA data

Primary alignments of mapped piRNA reads were imported as genomic ranges into R using function PICBload (R Bioconductor package PICB ^19,50^) for subsequent analyses and visualizations. piRNAs were annotated based on their overlap with protein-coding genes (RefSeq) and transposable elements (RepeatMasker). To avoid inflating read counts for piRNAs overlapping multiple genomic features, an annotation hierarchy was applied, prioritizing transposon annotations over protein-coding genes, antisense transposons over sense transposons, and sense protein-coding features over antisense: Transposon antisense (TE/as) > Transposon sense (TE/s) > CDS/s > 3′ UTR/s > 5′ UTR/s > INTRON/s > CDS/as > 3′ UTR/as > 5′ UTR/as > INTRON/as. Reads that did not overlap with any of these elements were assigned to “OTHER”. Sequence preferences of piRNAs and flanking genomic regions were visualized using the geom_logo function from the R package ggplot2 ^51^. To determine whether BFF captures piRNAs from the same genomic loci as standard protocols, we examined overlap with the coordinates of previously assembled mouse and *Drosophila* piRNA clusters ^19^ using Genomic Ranges (R Bioconductor) and plotted their cumulative abundance across the top clusters. Genome browser-style mapping over selected genomic loci was visualized as average piRNA coverage per 50-nucleotide window using ggplot2. Comparison between standard method-generated and BFF-generated Piwi-piRNA population sizes was performed after normalizing piRNA abundance in each dataset to the number of its 24-nt *flamenco* piRNAs.

### Transposon analysis of germline and follicle-cell Piwi-piRNAs

Follicle-specific and germline-specific Piwi-piRNA datasets were mapped to Repbase ^52^ to enable individual transposon-level analysis, including differential representation comparisons and strand bias analysis (see Supplementary Fig. 4d). Mapping was performed using STAR with the same settings that were used for mapping to the Drosophila genome (Dm6): allowing up to 1 mismatch, 100 alignments per read, and suppressing spliced reads. Differential transposon representation analysis was performed using the R package DESeq2 ^53^. The strand bias was visualized for transposons that were significantly enriched in one or the other dataset (≥ 4-fold enrichment (log_2_ fold-change > 2 or < − 2)).

### Coverage dispersion

We quantified coverage dispersion across genomic loci to measure how unevenly piRNAs are distributed within each locus. We used two separate measures: Coefficient of Variation (CV = SD(coverage_values)/mean(coverage_values), where CV = 0 if locus is uniformly covered) and Peak to Average Ratio (PAR = max(coverage_values)/mean(coverage_values), where PAR = 1 if locus is uniformly covered). As input, we used the average normalized number of piRNA reads per 50 nt bin throughout the selected loci. For the systematic investigation of PAR values across the top 80 MIWI clusters (producing 90% of piRNAs), we used only uniquely-mapping piRNAs and excluded 50 nt bins overlapping precursor introns, as these regions naturally lack coverage and would inflate dispersion measurements.

### Reproducibility of piRNA coverage patterns

To assess piRNA pattern/landscape conservation across replicates, we used the deepTools suite ^54^. First, we created normalized coverage files using bamCoverage with filtered bam files containing only primary alignments as input (bamCoverage -b rep1.bam --normalizeUsing CPM -o rep1.bw). By default, the above returns the normalized numbers of piRNA reads per 50 nt bin throughout the genome. The germline Piwi dataset was filtered for uniquely mapping reads only. We then used multiBigwigSummary with varying bin sizes (1 kb to 50 bp) to compute average coverage scores per bin throughout selected genomic loci: flamenco unique section for OSC and follicle-Piwi datasets (chrX:21,631,891–21,640,020); the 42AB locus for germline-Piwi datasets (chr2R:6,256,844–6,499,214); pi18 (chr18:67,028,116–67,074,900) and pi17 (chr17:27,284,000–27,372,300); and across the top 200 clusters (see Supplementary Data 2) for mouse MIWI datasets (see Figs. 5 and 7 and Supplementary Fig. 7). Pairwise Spearman correlations between replicates were calculated. Individual 5′ end analysis was performed in R as follows: genomic alignments were trimmed to their 5′ positions, and normalized per-nucleotide coverage within selected genomic loci was compared between replicates using pairwise Spearman correlation.

### Analysis of piRNA processing patterns

To search for regularly spaced piRNAs in our datasets, we plotted piRNA 5′ ends within 200 nucleotides surrounding the most productive processing site within selected loci (the *flamenco* unique section for unique OSC and follicle-Piwi piRNAs, and a shortened 42AB section (chr2R:6,414,700–6,424,700) for primary germline-Piwi piRNAs). We then extended this analysis to multiple highly productive, well-mappable genomic loci using the function *PICBbuild* (R Bioconductor package PICB ^50^) to extract regions with dense, unambiguous piRNA mapping (“seeds”) (see Supplementary Data 3–5). Seeds were filtered based on the following criteria: (1) at least 50% of the region covered by uniquely mapping reads for OSC and follicle-Piwi (40% for germline-Piwi, with the additional requirement of overlapping the 42AB locus, chr2R:6,256,844–6,499,214), (2) absence of multi-mapping reads (for OSC and follicle-Piwi only), and (3) presence in all biological replicates. This filtering yielded 83 seed regions for OSC (spanning 450,555 nt), 50 for follicle-Piwi (168,175 nt), and 20 for germline-Piwi (221,130 nt). For each filtered seed, we identified the most abundant 5′ end position, counted all 5′ ends within ± 100 nt of this position, and averaged the per-position counts across all seeds. Inset length distributions correspond to reads mapping to the most abundant 5′ end per region. In addition, to investigate 3′-to-5′ end relationships, we plotted the frequency of observed piRNA 5′ ends and 3′ ends surrounding each 5′ end position. Even though 3′-to-5′ end proximity cannot establish that the corresponding piRNAs originate from the same precursor transcript, it is sometimes cited as evidence of head-to-tail piRNA production. To assess whether 3′ end enrichment immediately upstream of 5′ ends reflects Zucchini’s sequence preferences, we compared distributions around 1U versus non-1U 5′ ends.

### Statistical analysis

Biological replicate numbers are reported in the corresponding figure legends. Data are shown as mean ± s.e.m. where indicated. Reproducibility of piRNA coverage profiles across biological replicates was assessed using Spearman rank correlation at the indicated genomic resolutions. Differential transposon representation between follicle-cell and germline Piwi-piRNA datasets was analyzed with DESeq2.

## Supplementary Material

This is a list of supplementary files associated with this preprint. Click to download.

- TableS4.xlsx
- TableS2.xlsx
- TableS1.xlsx
- TableS3.xlsx
- 2SupplementaryInformation.pdf
- SupplData1WBFig3b.xlsx
- nrsoftwarepolicy.pdf
- nrreportingsummaryBFF.pdf

## Figures and Tables

**Figure1:**
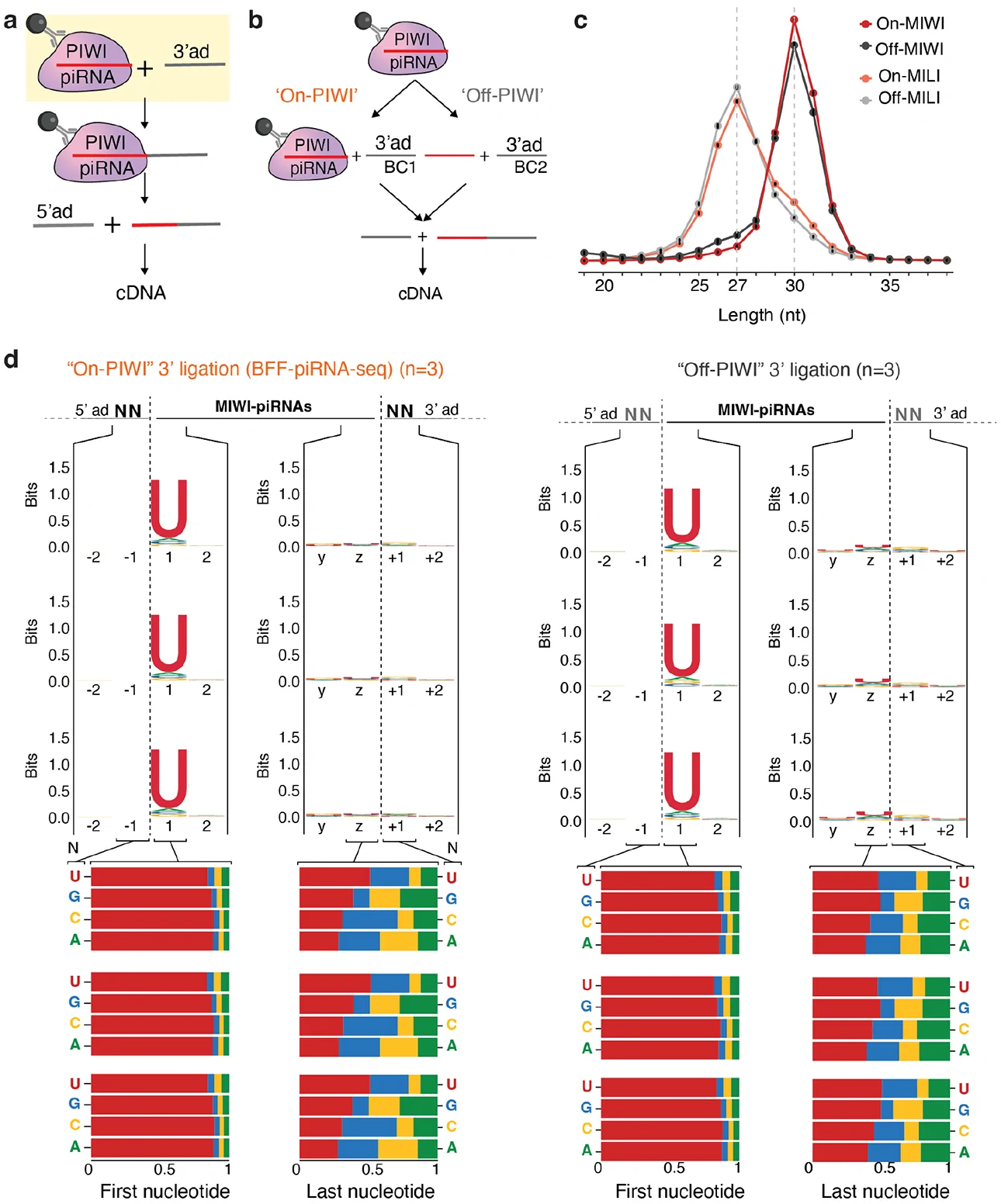
On-PIWI 3′ adapter ligation in BFF-piRNA-seq introduces no detectable length or sequence bias. (a) Schematic of BFF-piRNA-seq highlighting its key innovation, on-PIWI adapter (ad) ligation. (b) Experimental design used to assess potential length and sequence bias during the on-PIWI 3′ adapter ligation step. MIWI- and MILI-piRNA complexes were immunopurified from adult mouse testes and split for on- versus off-PIWI 3′ adapter ligation. For on-PIWI ligation, 3′ adapters were ligated directly to PIWI-bound piRNAs. As a control, piRNAs were first eluted from PIWI–piRNA complexes and then subjected to 3′ ligation in solution. On- and off-PIWI ligation reactions used barcoded 3′ adapters, allowing samples to be pooled for all subsequent steps, including 5′ adapter ligation, reverse transcription, and PCR amplification. Three biological replicates were generated using testes from three individual mice (n = 3). (c) Length distributions of MIWI- and MILI-bound piRNAs recovered after on-PIWI or off-PIWI 3′ adapter ligation show highly consistent profiles across three independent experiments (mean ± s.e.m.; n = 3). (d) Nucleotide frequencies at the 5′ and 3′ termini of MIWI-bound piRNAs recovered by BFF-piRNA-seq reveal the expected enrichment for uracil at the first nucleotide (1U bias). Random nucleotides (N) at the ligation junctions were incorporated to promote unbiased length and sequence representation. Nucleotide frequencies associated with individual terminal adapter nucleotides are shown below. Although ligation efficiency varies somewhat depending on the identity of terminal adapter nucleotides, the use of randomized terminal nucleotides prevents detectable ligation bias in both on-PIWI and off-PIWI ligation conditions.

**Figure 2:**
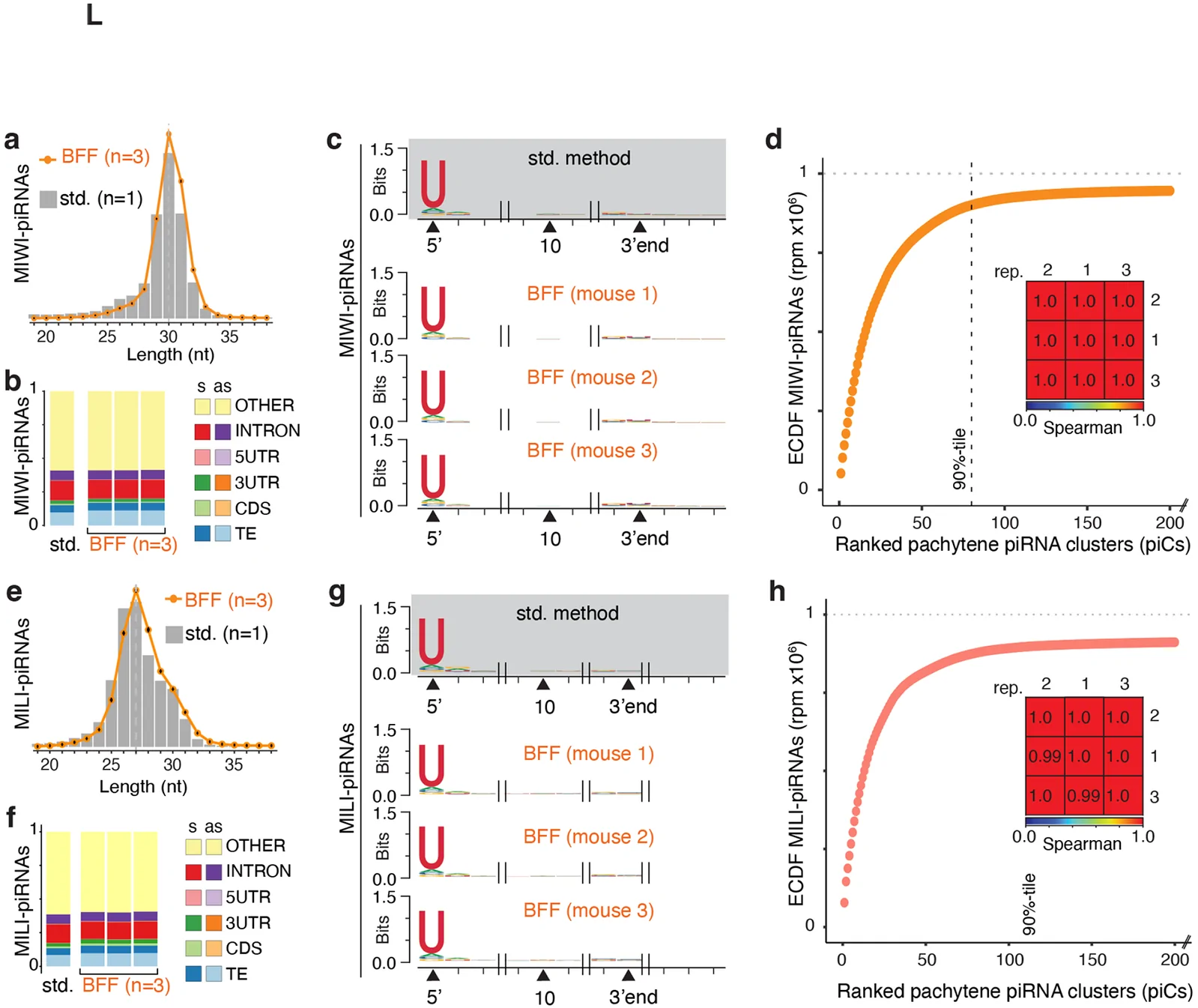
BFF-piRNA-seq faithfully recovers the defining features of MIWI- and MILI-bound piRNA populations. Comparison of BFF-piRNA-seq datasets for MIWI-bound (a–d) and MILI-bound (e–h) piRNAs with a standard reference dataset shows concordance in (a and e) length distributions, (b and f) genomic annotations, (c and g) 5′ uracil enrichment (1U bias), and (d and h) origin from piRNA-producing genomic intervals (piRNA clusters). These features were highly reproducible across three biological replicates (Spearman correlation, n = 3). TE, transposable element; UTR, untranslated region; ECDF, empirical cumulative distribution function.

**Figure 3:**
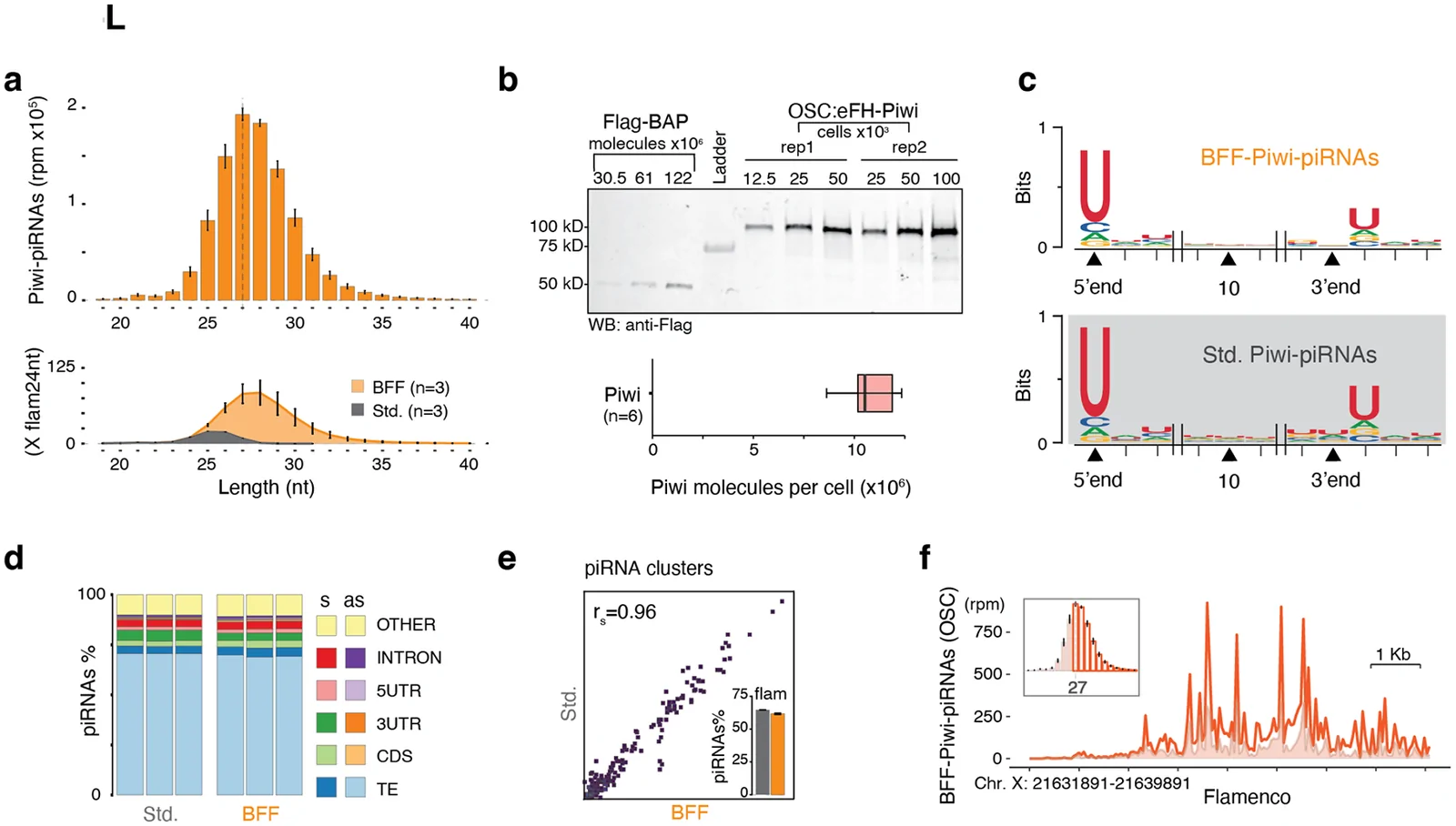
BFF-piRNA-seq reveals more and longer Piwi-piRNAs in ovarian somatic sheath cells (OSC). (a) Length distribution of BFF Piwi-piRNAs from OSC (mean ± s.e.m.; n = 3). The lower panel compares BFF and a standard protocol, each normalized to 24-nt flamenco piRNAs (mean ± s.e.m.; n = 3). (b) Quantification of Piwi protein in OSC: immunoblotting of FLAG-BAP standards and endogenously tagged FLAG-HA Piwi (eFH-Piwi) (top); estimated Piwi molecules per cell (bottom; n = 6). (c) Sequence logos for Piwi-piRNAs and downstream genomic context. (d) Genomic annotations of Piwi-piRNAs prepared by BFF versus the standard method (n = 3). (e) Spearman correlation (r_s) of piRNA abundance across clusters (90th percentile). The inset shows flamenco piRNAs (mean ± s.e.m.; n = 3). (f) Distribution of short (<27 nt) and long (≥27 nt) Piwi-piRNAs across the 5′ region of flamenco (chrX:21,631,891–21,640,020).

**Figure 4:**
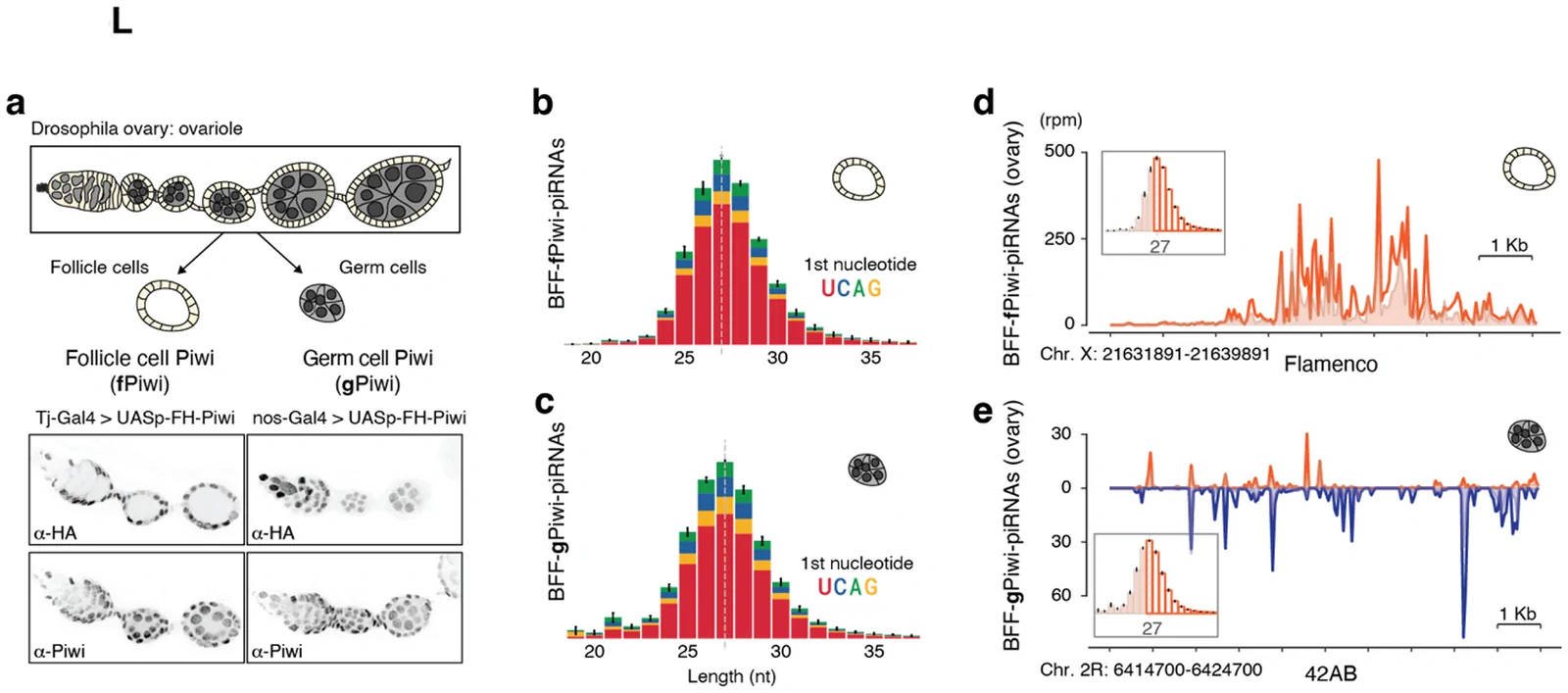
BFF-piRNA-seq reveals longer Piwi-piRNAs in ovarian soma and germ cells in Drosophila melanogaster. (a) Ovariole schematic and tissue-specific expression of FLAG–HA Piwi: follicle cells (tj>Gal4 and UASp-FlagHA(FH)-Piwi; follicle-cell Piwi, fPiwi) and germline (nos>Gal4 and UASp-FlagHA(FH)-Piwi; germ-cell Piwi, gPiwi). Anti-HA immunofluorescence; endogenous Piwi is shown as a control. (b,c) Length distributions of Piwi-piRNAs in follicle cells (b) and germ cells (c), with first-nucleotide preferences by length (mean ± s.e.m.; n = 3). (d,e) Distribution of short and long Piwi-piRNAs across representative intervals in flamenco (d, chrX:21,631,891–21,640,020; fPiwi) and cluster 42AB (e, chr2R:6,414,700–6,424,700; gPiwi).

**Figure 5:**
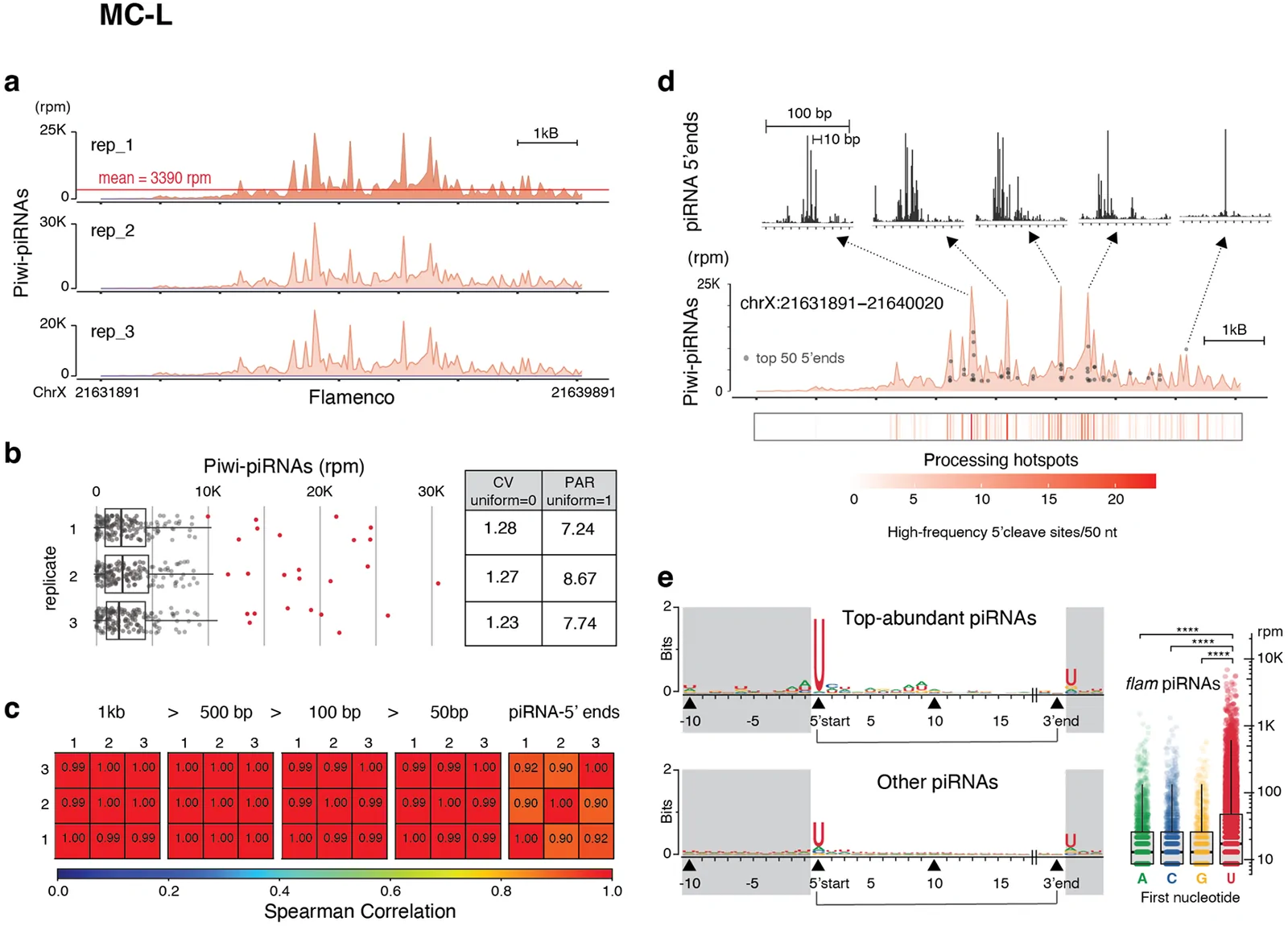
BFF-piRNA-seq reveals robust piRNA processing patterns marked by solitary hotspots. (a) Piwi-piRNA coverage across the 5′ region of flamenco (chrX:21,631,891–21,640,020) in OSCs (n = 3; 50-nt bins). The red line indicates mean coverage for a representative replicate. (b) Dispersion of coverage across the same interval, quantified in 50-nt bins as the coefficient of variation (CV; 0 = uniform) and peak-to-average ratio (PAR; 1 = uniform). (c) Reproducibility of coverage across biological replicates (Spearman r; n = 3), calculated at multiple resolutions ranging from 1 kb to single-nucleotide 5′ start sites. (d) piRNA 5′ start positions within selected coverage spikes (black bars, top). Gray dots indicate the 50 most abundant start sites and their normalized abundances. The lower panel shows heatmap clustering of the top 5% most frequently used 5′ start sites (n = 407) within 50-nt windows. (e) Sequence logos of Piwi-piRNAs derived from the 50 most frequent start sites (top left) versus all other start sites (bottom left). The right panel shows piRNA abundance by first-nucleotide identity, normalized to total flamenco piRNAs.

**Figure 6:**
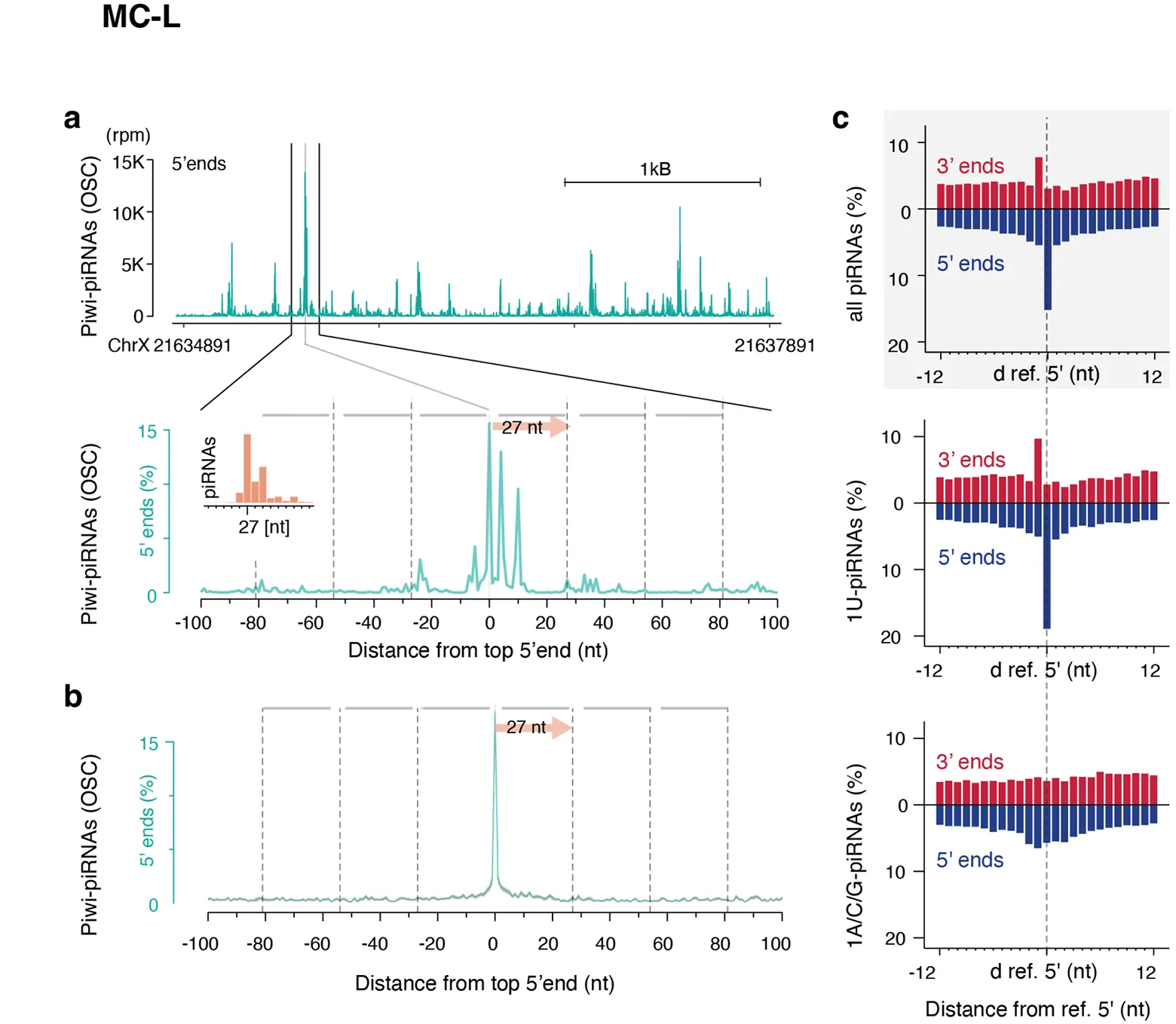
piRNA processing hotspots are solitary and give rise to locally confined piRNA populations. (a) Closeup of piRNA 5′ start sites centered on the most productive processing site in flamenco. The inset shows the length distribution of piRNAs across the displayed region, with the modal length indicated by the orange arrow. The lower panel shows the average distribution of piRNA 5′ start sites across three biological replicates centered on the most productive sites within uniquely mapping piRNA-cluster segments (n = 83). (b) Aggregate distributions of piRNA 5′ and 3′ ends relative to dominant 5′ start sites for all piRNAs, 1U piRNAs, and non-1U piRNAs across the same regions. (c) Positional relationship between piRNA 5′ and 3′ ends for all piRNAs, 1U piRNAs, and non-1U piRNAs.

**Figure 7:**
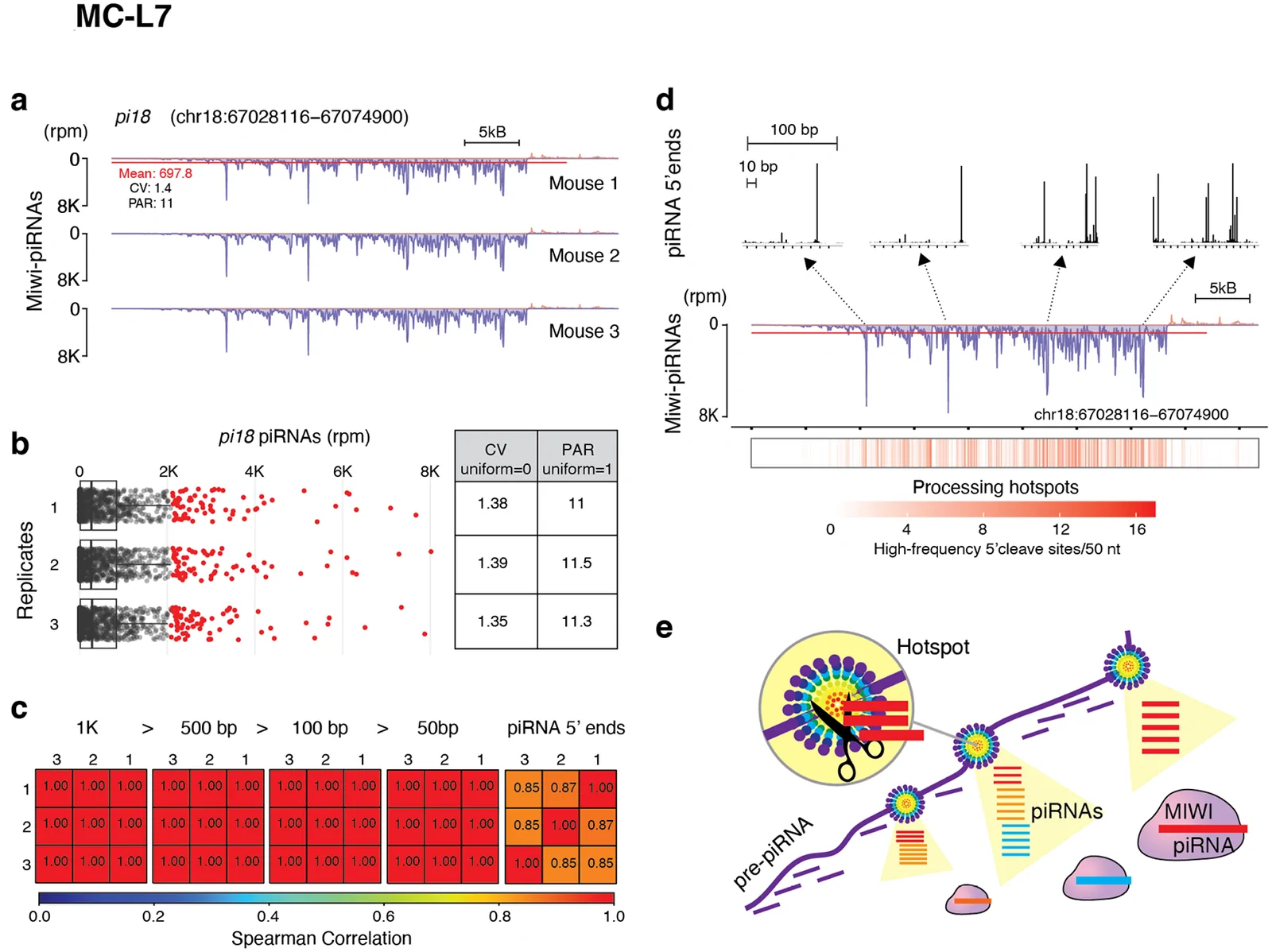
Highly abundant pachytene piRNAs arise from narrow processing hotspots. (a) BFF-piRNA-seq coverage of MIWI-bound piRNAs across the most productive pachytene piRNA cluster, pi18 (chr18:67,028,116–67,074,900; 50-nt bins; n = 3). The red line indicates mean coverage for a representative mouse. (b) Non-uniformity of piRNA coverage across pi18, quantified in 50-nt bins by the coefficient of variation (CV; 0 = uniform) and peak-to-average ratio (PAR; 1 = uniform). (c) Reproducibility of piRNA coverage across three mice, assessed at multiple resolutions ranging from 1-kb bins to individual 5′ start sites (Spearman r_s; n = 3). (d) piRNA 5′ start positions within processing hotspots (black bars, top). The lower panel shows the density of distinct high-frequency 5′ start sites (top 5%) within 50-nt windows, highlighting clustered and highly preferred precursor positions (n = 2,325; top 5% of pi18 5′ ends). (e) Model for piRNA biogenesis in pachytene clusters. piRNAs are generated preferentially from discrete hotspots along long piRNA precursors, with one or a few favored cleavage sites within each hotspot defining the 5′ ends of highly abundant piRNAs.

## Data Availability

Sequencing data generated in this study are deposited in the Gene Expression Omnibus under accession GSE313113 and will be made publicly available upon publication. All data are available to reviewers via the following private access token: qlotugquvnwhzof. https://www.ncbi.nlm.nih.gov/geo/query/acc.cgi?acc=GSE313113

## Code availability

All custom code used in this study is provided as Supplementary Software and will be made publicly available upon publication. The final version will be released through GitHub and archived in Zenodo upon publication.
